# Exostosis

**Published:** 1854-07

**Authors:** S. H. Harvey

**Affiliations:** McConnellsburg, Pennsylvania.


### ARTICLE VII.

*Exostosis.* Letter from Dr. S. H. HARVEY, McConnellsburg, Pennsylvania.

ENCLOSED I send you three teeth, which I extracted from the mouth of Mrs. L., of this place. Her health very delicate, slightly disposed to scorbutic, had suffered very much from her teeth, the salivary glands constantly discharging very freely. Mrs. L. had thirteen teeth taken out at one sitting, all of which were more or less effected with exostosis. The three I send you, however, are the most strongly marked. She suffered constant pain, and her health gradually appeared to grow more and more feeble. But from her extreme timidity and dread of the operation, she resisted for a long time, her own good sense, the advice of her family physician and the wish of her husband, to the extraction.

The teeth I send you are the left superior cuspidatus and bicuspids. There was a thin purulent and offensive discharge of pus followed the extraction of the most marked cases. I am happy to add, the health of Mrs. L. has very materially improved, and is still improving since the extraction, and she confidently now believes she again will be restored to her wonted good health.

NOTE.—The senior editor begs to return his thanks to Dr. S. H. Harvey, for the specimens of dental exostosis mentioned in the above letter. He will have them placed with others in the Museum of the Baltimore College of Dental Surgery, where they may be examined by professional gentlemen visiting the institution.

